# Letters from South American Surgeons on Cundurango, to E. Andrews, M.D., Professor of Surgery in Chicago Medical College

**Published:** 1872-05-01

**Authors:** 


### Original Communications.

### LETTERS FROM SOUTH AMERICAN SURGEONS ON CUNDURANGO, TO E. ANDREWS, M.D., PROFESSOR OF SURGERY IN CHICAGO MEDICAL COLLEGE.

In November last I wrote to a number of prominent physicians and surgeons in Ecuador, asking their opinions and their facts on the efficacy of this new drug.

The postal communications with that country are so slow that I have only recently received replies. As I read Spanish with some difficulty, I have availed myself of the kindness of Mr. John Sundberg, of the College class, who is more familiar with that language, and who has made the subjoined translations.

The first letter is from the Surgeon-in-Chief of the Army of Ecuador, Dr. Oblonoxato Auriboje, and is as follows:

GUAYAQUIL, Jan. 12, 1872.

*Senor Dr. Edmund Andrews:*

DEAR SIR—I have the honor to answer your valuable letter of Nov. 15 last year. The reason why I did not do it by return steamer, was because I had been absent from Guayaquil for thirty days.

Concerning cundurango, I can assure you that it is a very efficacious remedy in the syphilitic affections of the mucous membrane; in several diseases of the skin, especially the herpetic; in several eczemas, and in most of the diseases which arise from impure blood.

In rheumatism it is a powerful recourse, especially in the fibrous and muscular forms, after first having suitably prepared the patient.

In cancerous diseases it is a powerful alterative—above all if the disease is not very old. Of cases of radical cure I only know two—one a cancer of the tongue, whose history you will see in the periodical "*El Nacional*," No. 100, and the other a case of cancer (not ulcerated) of the right breast—terminated successfully under the use of cundurango. Great improvements of ulcerated cancers are seen every day. To-day I have a case of an enormous cancer of the right breast. That the progress of the disease has stopped is indubitable. It is about eight months old. It seems as if the action of the medicine is more certain in young persons than in subjects of old age. In external diseases I make use of local applications of different preparations of cundurango. Internally I have used infusion, and experience has shown me that this is the best mode for use.

I shall have much honor in communicating in the future all relating to scientific works on cundurango.

Yours truly,

OBLONOXATO AURIBOJE, M.D.,

*Surgeon-in-Chief of the Army.*

From this epistle it appears that the distinguished writer values the remedy most for its anti-syphilitic power, but that he also has great confidence in its ability to cure some cases of cancer.

The second letter is from Dr. Camilo Casares, Professor of Surgery in the University of Quito. It will be seen that the Professor relied on his published documents, to be forwarded by the next steamer, to place me in possession of his observations. Unfortunately these have not yet arrived, so that I can only say, in general terms, that he appears to endorse the efficacy of cundurango. I have written again for the papers, and when they arrive will acquaint the readers of THE EXAMINER with their contents. Here is the letter:

QUITO, 27th of December, 1871.

*Senor Dr. Edmund Andrews, Professor of Surgery Chicago Medical College:*

DEAR SIR—I have the pleasure of answering your valuable letter, dated in Chicago on the 15th of November, 1871, assuring you that by the first steamer you will have all the data which I have been able to collect in the hospital which is in my charge, as well as in my private practice, in regard to the properties of cundurango. The many contradictory opinions which this plant has roused, has induced me to publish an article, if not scientific, at least with truth and sincerity, since so important a discovery can with difficulty be judged in the short time which this has had.

I am happy to avail myself of this opportunity to offer you my friendship.

Our communications, which no doubt from this time onward will be established, will be easy and secure by the favor of my much esteemed friend, Col. Rumsey Wing, American Minister in Ecuador.

I wish you had correspondence with Dr. Bliss, and you would deign to give him my respects. The rudeness and want of justice with which this gentleman has been treated in the *Times* for his opinions in regard to cundurango, have vividly excited my sympathy for him.

From your friend and obedient servant,

CAMILO CASARES,

*Professor of Anatomy and Surgery in the University of Quito, Ecuador.*

Fourth street, No. 36, Quito.

The third letter is from Dr. Augustin Ruiz, of Loja. This town, Loja, is the capital of a province of the same name in the heart of the Andes, and is in the region where the cundurango grows, and where its qualities were first experimented on. It will be seen that Dr. Ruiz offered to sell me the article, delivered in New York, at two dollars a pound, when Bliss & Keene were doling it out at forty dollars a pound. It will also be noticed that the old Indian in Loja who first took it, and is reported by Dr. Bliss to have been thereby cured of cancer, is stated by Dr. Ruiz to have had nothing but tertiary syphilis; and, further, that he is not aware that cundurango possesses any power to cure cancer. He is, however, like the other medical gentlemen of Ecuador, strongly impressed with its anti-syphilitic power.

LOJA, January 14, 1872.

*Prof. Edmund Andrews:*

DEAR SIR—I have received, on the 6th inst., your most valuable letter of November last year, wishing me to communicate to you my opinion of the efficacy of cundurango in the treatment of syphilis and cancer, together with some of the facts which have come under my own observation. In reply to this I shall state the following:

The cundurango was only known in this province as a poison, with which dogs were killed, until a woman—whose husband, fifty years of age, who was suffering with constitutional syphilis, from which almost his whole body, and especially his face, was ulcerated—wishing to get rid of him, as she was unable to support him, resolved to kill him with the plant in question, and began to give him a decoction of it under the pretext that it was a remedy which she had been advised to use for him. The poor old man took his first portion, which his wife hoped would settle him. But seeing that her object was not accomplished, and attributing the bad result to a too small dose, she continued to administer it to him daily. But to her great surprise, at the end of eight days, her husband commenced to get better rapidly, and at the end of one month he was completely cured. The husband, on the other hand, was also surprised at having recovered from a disease which he had had two or three years, and with which he had expected to die, and he asked his wife what the so miraculous remedy was which had produced in him such a famous cure. She disclosed to him that it was cundurango which she had given him, and which had done him so much good. This, sir, is the origin of this discovery. Since then the town, which was overflown with syphilis, has known this remedy, and it has never failed yet during a period of about forty years. I have used it for about six years in more than thirty cases of tertiary syphilis, and in none has it failed to produce a radical cure in less than forty days.

Chronic rheumatism, and some kinds of incipient paralysis dependent on the same, have been treated and cured by cundurango, and with good result. I have administered it in two cases of this disease, and in both obtained the most happy results.

As to its anti-cancerous action I can say nothing, as I have not tried it in this disease, no well confirmed case of this kind having ever presented itself to me. But let me tell you that this remedy acts as an alterative in all diseases of the skin, modifying and curing most of them. This is all what I, as a physician, have observed about cundurango.

The cundurango on which our observations have been made, is the one which grows in our province, Loja, although it is said to grow in other parts. As I, however, know only ours, I am unable to say whether the others possess the same medical virtues. We have only administered it in decoction. Cort. cundurango, ℥ ss to aqua Oj, boiled down to ℥ xjj. This decoction to be taken daily, divided into three draughts. After twelve days we have increased it to ℥ j of the bark, to the same quantity of water.

If you need a few quintals (100 lbs.), I shall be able to afford about 50 quintals of the genuine bark, sent to Guayaquil or to New York, at the price in Gnayaquil of $80, and in the United States of $100. If you need any, inform me when convenient to send.

Your most obedient servant,

Augustin Ruis.

The last letter is from Dr. Jose Maria Eguiguren, brother of the Governor of the province of Loja. Dr. Eguiguren was the first physician to experiment on the article, and though he afterwards abandoned his practice to go into political life, his reports were the means of causing Prof. Casares and Surg.-Gen. Auriboje to take up the investigation. The doctor says that the Indians, from whom he first obtained the remedy, use with it another plant, which, very powerfully increases its efficacy. Of this other plant he gives no description, and the specimens which he promised have not yet arrived.

Loja, February 3, 1872.

*Senor Dr. Edmund Andrews:*

Dear Sir—When at the receipt of your favor of last year, I did not answer it immediately, it was because I wanted to give you an exact account of all the observations in regard to cundurango which I had made in my practice. Know that it is the terrible whip which the human species has against the syphilitic diseases; likewise against the cancerous affections, in certain cases of which all the resources of the art have proved so inefficient; also, in diseases of a rheumatic character; in various neuralgias, otalgias, cutaneous diseases, and in many of those which arise from impure blood. Although it has proved a specific in so great evils, it nevertheless gives excellent results in others —for instance, in dysentery of a putrid character. Unfortunately, a criminal fraud has given discredit to this so famous plant. There have been introduced into the market others of various families in excessive quantity, and of much inferior quality to that which has been made use of in our observations. Like the cinchonas, it has been wished that all varieties should enjoy the same reputation. There are recognized in our regions five varieties of cundurango: 1st. The red, which, although very scarce, is superior in activity to all the others; 2d. The yellow, which also is very scarce; and the three others, which only differ by their fruit, are found in great quantities. As we owe this discovery to the natives of our soil, these have also presented us with a powerful help in the efficacy of the mode of using this plant, uniting it with an extract drawn from a bark called *oriental.* I do not doubt that your attention will be called to the miraculous mode of operating of this other substance. It is not only commendable for its emeto-cathartic action, but it is a purgative worthy of all confidence, and it has an admirable effect in the various diseases of the liver, stomach and intestines. Being an excellent purifier, when used in cases of impurity of the blood of a cancerous or syphilitic nature, it will lend an assistance so powerful that *he who administers so precious a vegetable, will never see his hopes fail.* Thus, also, when used in all the cases of diverse skin diseases, as these diseases seldom have any other origin than the disturbance of the different fluids of the human body. As a purifier, it frees the blood from all its impurities, and leaves a freshness to the skin, so that the patient has nothing more to wish. I should have much more to say if I should mention all which has come under my observation in my practice; but I wish you to form your own judgment over all that has been said, and give me the pleasure of receiving your letters.

I take this opportunity to offer you my friendship, and subscribe myself your most attentive and secure servant,

Jose Maria Eguiguren.

P.S.—I forgot to tell you that by the next mail I shall send you a small package of the bark of which I have spoken to you, and afterwards I shall send you for trial a seed of the plant whose virtues I have heard so much about. I have been told that it is a very active poison.

To sum up the result, it appears that the profession in Ecuador have confidence in cundurango, more especially in syphilis; but it is very doubtful whether the fluid extracts retailed to us are properly made from the genuine drug. In this country most of the experiments have been made in cancer, and though the result has not been perfectly satisfactory, we should not abandon the investigation until we have obtained an article known to be genuine, and tried it, not only in cancer, but also in syphilis.

I have written again to Prof. Casares, asking him to send a quantity of the article which he knows to be genuine.

No. 6 Sixteenth st., Chicago,
April 25, 1872.

